# Genomic Characterization of Cisplatin Response Uncovers Priming of Cisplatin-Induced Genes in a Resistant Cell Line

**DOI:** 10.3390/ijms22115814

**Published:** 2021-05-28

**Authors:** Hadar Golan Berman, Pooja Chauhan, Shira Shalev, Hiba Hassanain, Avital Parnas, Sheera Adar

**Affiliations:** Department of Microbiology and Molecular Genetics, Institute for Medical Research Israel Canada, Faculty of Medicine, Hebrew University of Jerusalem, Ein Kerem, Jerusalem 91120, Israel; hadar.golan@mail.huji.ac.il (H.G.B.); pooja.chauhan@mail.huji.ac.il (P.C.); shira.shalev1@mail.huji.ac.il (S.S.); hiba.hassanain@mail.huji.ac.il (H.H.); avital.parnas@mail.huji.ac.il (A.P.)

**Keywords:** cisplatin, cancer, resistance, DNA damage, DNA repair, nucleotide excision repair, chromatin, transcription

## Abstract

Cisplatin is a chemotherapy drug that kills cancer cells by damaging their DNA. In human cells, this damage is repaired primarily by nucleotide excision repair. While cisplatin is generally effective, many cancers exhibit initial or acquired resistance to it. Here, we studied cisplatin resistance in a defined cell line system. We conducted a comprehensive genomic characterization of the cisplatin-sensitive A2780 ovarian cancer cell line compared to A2780cis, its resistant derivative. The resistant cells acquired less damage, but had similar repair kinetics. Genome-wide mapping of nucleotide excision repair showed a shift in the resistant cells from global genome towards transcription-coupled repair. By mapping gene expression changes following cisplatin treatment, we identified 56 upregulated genes that have higher basal expression in the resistant cell line, suggesting they are primed for a cisplatin response. More than half of these genes are novel to cisplatin- or damage-response. Six out of seven primed genes tested were upregulated in response to cisplatin in additional cell lines, making them attractive candidates for future investigation. These novel candidates for cisplatin resistance could prove to be important prognostic markers or targets for tailored combined therapy in the future.

## 1. Introduction

Cisplatin (*cis*-diamminedichloroplatinum(II)) is a first-line chemotherapeutic drug broadly used in the treatment of multiple cancer types, including testicular, bladder, head and neck, cervical, and ovarian cancers [1]. After entry and activation in the cells by aquation, cisplatin reacts and binds purine bases in DNA, forming primarily di-adducts that distort the helical structure of DNA [2,3]. This cisplatin-induced DNA damage blocks transcription and replication and subsequently induces apoptosis in cancer cells. In human cells, this damage is repaired primarily by nucleotide excision repair (NER) [4,5,6,7].

The NER pathway [8,9] begins with the identification of a bulky DNA damage either directly by the excision repair factors or by a blocked RNA polymerase. The two different damage recognition mechanisms divide NER to two sub pathways: global genome repair (GGR) and transcription-coupled repair (TCR). After damage recognition, the subsequent steps of the two pathways are identical and include dual incisions of the damaged DNA strand, releasing an ~26 nt oligomer containing the damage, and leaving a short single stranded gap in the genome. This gap is then filled-in and ligated by a DNA polymerase and DNA ligase, using the undamaged strand as a template, resulting in an error-free repair [8,9]. Different types of damage are preferentially repaired by GGR or TCR. Mapping NER across the genome at single nucleotide resolution using XR-seq provides a precise tool for discriminating TCR and GGR patterns of repair [10,11,12]. When damage is removed primarily by TCR, repair is enriched at transcribed regions and preferential repair is observed in the transcribed strands of genes. In contrast, GGR which is not coupled to transcription does not exhibit this strand bias.

While therapy with cisplatin or its derivatives is generally effective, many cancers possess or develop resistance with time, resulting in recurrence [13]. Multiple pathways may lead to cisplatin resistance [14,15]. These include reducing the intracellular accumulation of the drug by either decreased uptake or increased efflux, and increased detoxification of the drug. The expression levels of proteins involved in these pathways have been linked to resistance. Copper transporter 1 (CTR1) was shown to increase cisplatin uptake, and the expression levels of the copper-extruding P-type ATPases, ATP7A and ATP7B, are associated with resistance [16]. Similarly, expression of the MRP2 enzyme, implicated in cisplatin efflux, negatively correlates to drug response [17,18,19]. Another enzyme that promotes resistance is glutathione S-transferase, which is involved in drug sequestration and inactivation [20,21,22]. Deficient NER sensitizes cells to cisplatin, and therefore, enhanced repair could contribute to resistance [15,23,24].

Here, we used comprehensive genomic characterization of a cisplatin-resistant cell culture model in order to identify possibly key factors in resistance. We found that resistance in these cells is obtained by lower DNA damage formation, without a significant change in repair rates. We identified a list of genes upregulated in the sensitive cells upon cisplatin treatment, that are already primed in the resistant cells, and that are promising novel candidates for cisplatin resistance.

As a model for cisplatin resistance, we used the p53 proficient A2780 ovarian cancer cell line [25], which is sensitive to cisplatin, and A2780cis, from here referred to as “Acis” cells, which was created after chronically exposing A2780 cells to increasing concentrations of cisplatin to create a cisplatin-resistant cell line (Supplementary Figure S1, [26]).

### 1.1. Lower Cisplatin Damage Formation but Similar Repair Kinetics in Acis Compared to A2780 Cells

**Figure 1:**
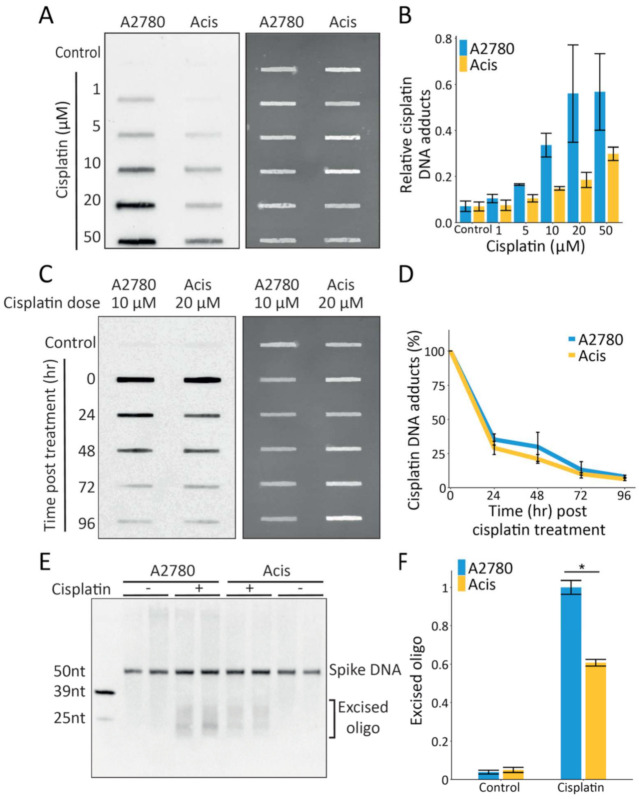
Lower cisplatin damage formation but similar repair kinetics in Acis compared to A2780 cells. (**A**) Representative immuno-slot blot (left panel) for cisplatin initial damage following increasing cisplatin doses treatment (1 µM–50 µM). Total nucleic acid amounts measured by SYBR-Gold staining (right panel). (**B**) Damage signal normalized to total nucleic acid amounts, showing cisplatin initial damage signal following increasing doses. Averages and error bars (SEM) are shown for data from three biological replicates. (**C**) Representative immuno-slot blot for cisplatin damage signal at different time points following cisplatin treatment (left panel). Total nucleic acid amounts measured by SYBR-Gold staining (right panel). (**D**) Damage signal normalized to total nucleic acid amounts, showing cisplatin repair kinetics of A2780 and Acis cell lines. Averages and error bars (SEM) are shown for data from two biological replicates. (**E**) Representative blot of excised oligos released during NER following 200 µM cisplatin treatment in A2780 and Acis cells. (**F**) Normalized excision products calculated by dividing excision product signal by 50 nt spike- in DNA amounts. Data are represented as mean ± SEM from three biological replicates. * *p* < 0.05 based on paired Student’s *t*-test.

From a DNA damage perspective, cisplatin resistance could be obtained either by lower initial damage levels or enhanced repair. To test damage formation, A2780 and Acis cells were treated with increasing doses of cisplatin and their genomic DNA was extracted and subjected to immuno-slot-blot analysis. Acis cells consistently exhibited lower initial damage levels compared to A2780 cells across low (1–50 µM) and high (50–200 µM) cisplatin doses (Figure 1A,B and Supplementary Figure S2). At very high doses (500 µM) the differences between the damage levels in the two cell lines diminished.

To test repair kinetics, cells were allowed to repair DNA after damage treatment and prior to DNA extraction and immuno-slot blot. Since cisplatin repair is relatively slow and requires several days, we assayed repair under conditions that allowed at least 50% A2780 cell survival. Due to the difference in damage formation between the sensitive and resistant cell lines, A2780 and Acis cells were treated with 10 µM and 20 µM cisplatin, respectively, to produce similar initial damage levels. Immuno-slot blot analysis of damage levels across time showed that A2780 and Acis cells exhibit similar repair kinetics (Figure 1C,D).

While immuno-slot blotting measures repair based on damage removal, the excision assay allows direct measurement of repair based on quantification of the excised oligo released during repair. This assay requires relatively high treatment doses and was performed 3 h after treatment of both cell lines with 200 µM cisplatin. Higher repair (~1.6-fold more excised oligos released) was observed in the sensitive A2780 cells compared to Acis cells (Figure 1E,F). However, this higher repair could be a product of the 1.4-fold higher level of damage observed in A2780 cells after treatment with 200 µM cisplatin (Supplementary Figure S2).

**Figure 2:**
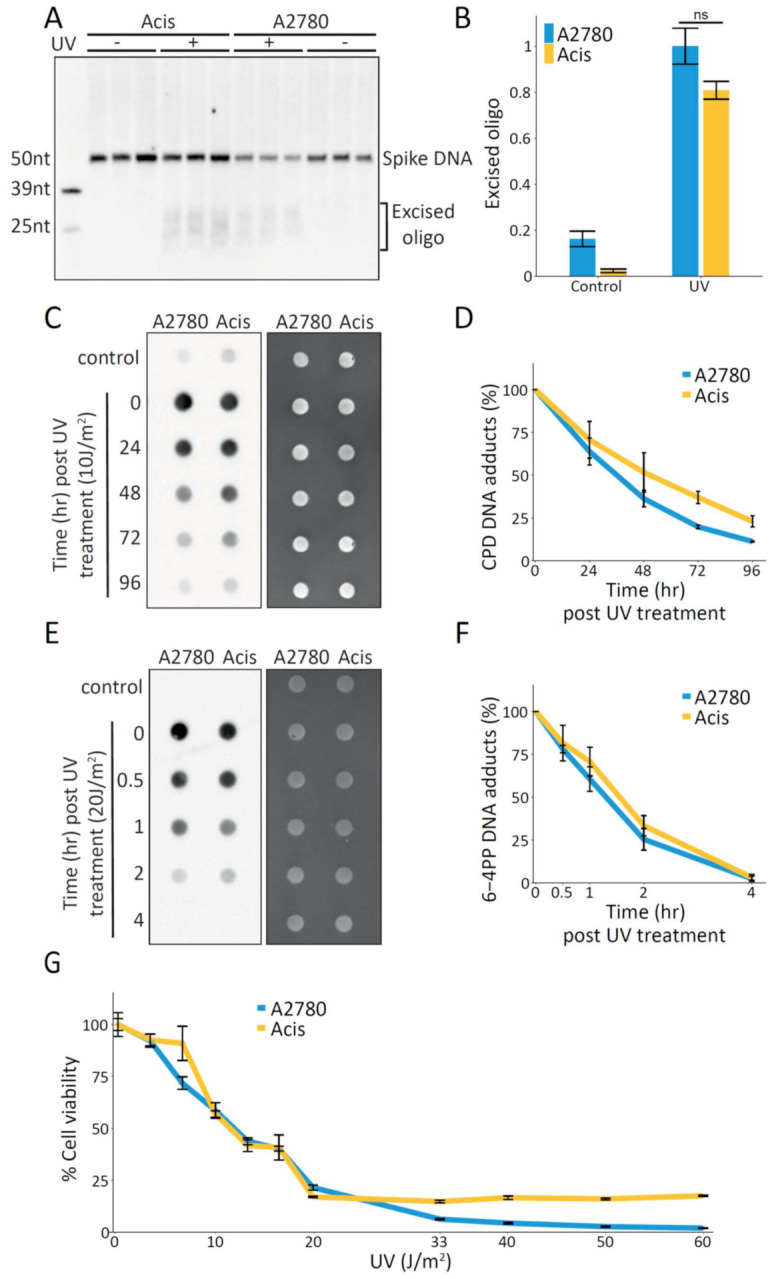
Similar UV damage repair kinetics in A2780 cells compared to Acis cells. (**A**) Representative blot of excised oligos released during NER following 20 J/m2 UVC irradiation in A2780 and Acis cells. (**B**) Normalized excision products calculated by dividing excision product signal by 50 nt spike- in DNA amounts. Data are represented as mean ± standard error from three biological replicates. *P* value based on paired Student’s *t*-test. (**C**) Representative immuno-slot blot for CPD damage signal at different time points following UV treatment (left panel). Total nucleic acid amounts measured by SYBR-Gold staining (right panel). (**D**) Damage signal normalized to total nucleic acid amounts, showing CPD repair kinetics of A2780 and Acis cell lines. Averages and error bars (SEM) are shown for data from four biological replicates. (**E**) Representative immuno-slot blot for 6-4PP damage signal at different time points following UV treatment (left panel). Total nucleic acid amounts measured by SYBR-Gold staining (right panel). (**F**) Damage signal normalized to total nucleic acid amounts, showing 6-4PP repair kinetics of A2780 and Acis cell lines. Averages and error bars (SEM) are shown for data from five biological replicates. (**G**) Cell viability of A2780 and Acis cell lines following increasing UV doses.

In contrast to cisplatin damage formation, which is influenced by cellular factors such as import, efflux, and detoxification, UV dimer formation is almost instantaneous. To isolate the kinetics of nucleotide excision repair independently of the kinetics of damage formation, we measured the repair of UV damage. Similar to cisplatin repair, measurement of UV-induced repair by the excision assay showed slightly (but not significantly) higher repair in A2780 cells compared to Acis cells (Figure 2A,B). Since in the excision assay we did not differentiate between the two major UV-induced types of damage, cyclobutene pyrimidine dimers (CPDs) and pyrimidine (6-4) pyrimidone photoproducts (6-4PPs), we also performed immuno-slot-blot analysis for both these types of damage. CPD repair kinetics was slightly higher in A2780 cells compared to Acis cells (Figure 2C,D), while 6-4PP repair kinetics was similar in both cell lines (Figure 2E,F). Most importantly, it does not appear that Acis cells have increased resistance to UV damage (Figure 2G). Together, these results indicate the cisplatin-resistance phenotype of Acis cells cannot be attributed to improved NER.

### 1.2. Comprehensive Genomic Characterization of the Cisplatin-Resistance Model

**Figure 3:**
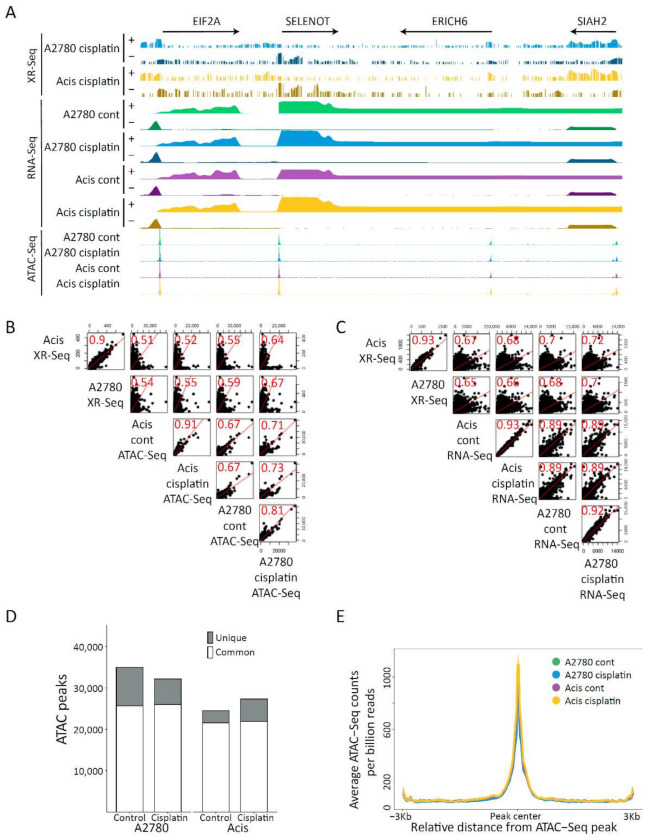
Comprehensive genomic characterization of a cisplatin-resistance model. (**A**) Repair, expression, and accessibility distribution of A2780 and Acis cells treated with 200 µM cisplatin over ~260 Kb region of chromosome 3 (150,535,931–150,800,463). Arrows on the top depict the direction and length of annotated genes. (**B**) Scatter plots showing Spearman correlation between cisplatin repair signal and accessibility levels over ATAC peaks. (**C**) Scatter plots showing Spearman correlation between cisplatin repair signal and transcription levels over genes. (**D**) ATAC common and unique peaks for each cell line before and after cisplatin treatment. (**E**) Accessibility signal over A2780 control ATAC-Seq peaks shared by all conditions. The data represent the average of biological replicates with a bin size of 30 nt.

To identify differences in the genomic response to cisplatin-induced DNA damage, we profiled NER activity, transcription, and chromatin accessibility in cisplatin-sensitive and -resistant cells treated or untreated with cisplatin. We used XR-seq to map repair at single nucleotide resolution. Cells were incubated with cisplatin for 3 h, allowing sufficient time for damage induction and repair to begin. Following this, the excised oligos released during NER were captured and sequenced and cisplatin excision repair maps were created for each strand separately (Figure 3, Supplementary Figure S3). In parallel, total stranded RNA-seq and ATAC-seq [27] were performed under the same conditions in cisplatin-treated and untreated A2780 and Acis cell lines to, respectively, establish differences in transcription and chromatin accessibility. In general, A2780 and Acis cell lines showed very similar repair, transcription, and chromatin accessibility profiles (Figure 3A, Supplementary Figure S4). Consistent with previous findings [11,28], the cisplatin repair signal positively correlated with both transcription levels at genes and with accessibility level at ATAC-seq peaks (Figure 3B,C). Cisplatin treatment did not result in a large shift of accessibility. Compared to 34,979 initial ATAC-seq peaks in untreated A2780 cells, cisplatin treatment resulted in a loss of 9329 peaks and a gain of only 6152 peaks (Figure 3D). The ATAC-seq signal over shared peaks between cisplatin-treated or untreated A2780 and Acis cells did not show a gain or loss of accessibility between the two cell lines or after treatment (Figure 3E).

### 1.3. A Shift in the Balance of TCR vs. GGR in Acis Cells Compared to A2780 Cells

**Figure 4:**
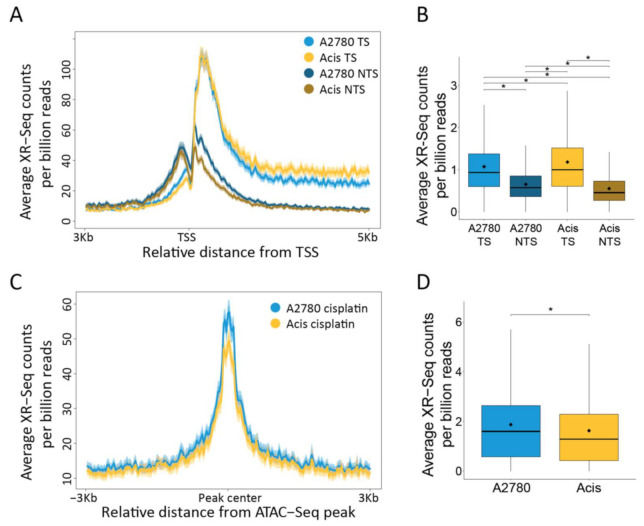
A shift in the balance of TCR vs. GGR in Acis cells compared to A2780 cells. (**A**) Cisplatin XR-seq repair signal 3 Kb upstream and 5 Kb downstream of the annotated TSS of 2192 genes. Signal is plotted separately for the transcribed and non-transcribed strands. The data represent the average of three biological replicates for A2780 cells and two for Acis cells with a bin size of 40 nt. Shadow represent 95% confidence interval for the mean. (**B**) XR-Seq read counts over 2192 genes in the transcribed and non-transcribed strands. * *p* < 0.0001, based on Wilcoxon signed-rank test with Bonferroni correction. Boxes represent range between 75th and 25th percentile, the line represents the median and the diamond the mean. (**C**) Cisplatin XR-seq repair signal over ATAC-Seq peaks and 3 Kb flanking regions with a bin size of 30 nt. Shadow represent 95% confidence interval for the mean. (**D**) XR-Seq read counts over ATAC-Seq peaks. * *p* < 0.0001, based on Wilcoxon signed-rank test with Bonferroni correction.

Cisplatin repair profiles over annotated genes in both cell lines indicate preferential repair by TCR evident by a pronounced bias towards repair of transcribed regions (Figure 3A) and the transcribed strand (Figure 4A,B). While repair was generally similar between A2780 and Acis cells, the balance between TCR and GGR appears to be altered. Acis cells exhibited a stronger bias than A2780 cells for transcribed strand repair. In contrast, cisplatin repair profiles at ATAC-seq accessibility peaks in non-transcribed regions was slightly elevated in A2780 cells (Figure 4C,D), indicating GGR was elevated in A2780 cells compared to Acis cells. We conclude that in Acis cells there is a shift in the balance between the two damage recognition pathways and TCR becomes more prominent.

### 1.4. Gene Expression Analysis Identifies Cisplatin-Responsive Genes Primed in Acis Cells Prior to Treatment

**Figure 5:**
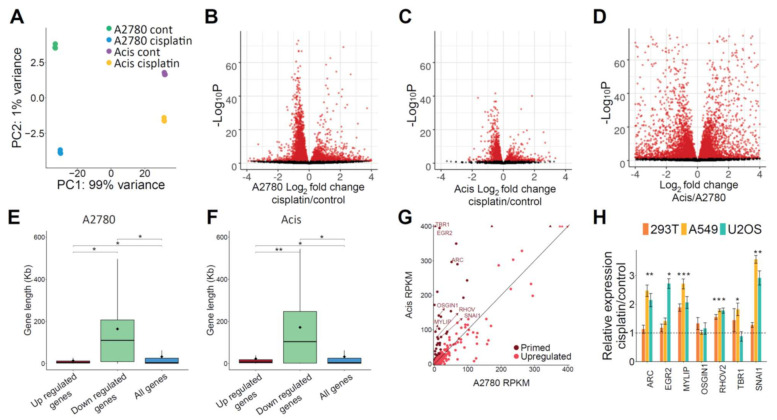
Gene expression analysis identifies cisplatin-responsive genes primed in Acis prior to treatment. (**A**) PCA plot of A2780 and Acis control and cisplatin-treated RNA-seq libraries. (**B**) Volcano plot for differentially expressed genes in A2780 control vs. cisplatin treated samples. (**C**) Volcano plot for differentially expressed genes in Acis control vs. cisplatin-treated samples. (**D**) Volcano plot for differentially expressed genes in A2780 control vs. Acis control. (**E**) Gene length distribution of differentially expressed genes in A2780. Gene length distribution of all annotated genes is shown for comparison. * *p* < 0.0001, based on Wilcoxon rank sum test. (**F**) Gene length distribution of differentially expressed genes in Acis. * *p* < 0.0001, ** *p* < 0.001 based on Wilcoxon rank sum test. (**G**) A2780 and Acis control RPKM levels of upregulated genes following cisplatin treatment in A2780 cells. Genes included in the primed genes list are colored in dark red. RPKM levels higher than figure limit are depicted using arrowheads. (**H**) Real-time quantitative PCR relative expression levels of candidate genes in 293T, A549, and U2OS cell lines following cisplatin treatment. Values represent averages of four biological replicates ± SEM. * *p* < 0.05, ** *p* < 0.01, *** *p* < 0.001 based on paired Student’s *t*-test.

Consistent with cisplatin adducts blocking RNA polymerases, analysis of differentially expressed genes after cisplatin treatment identified more downregulated than upregulated genes (Figure 5A–C). We do not observe such differences in comparing expression between the sensitive and resistant cell lines (Figure 5D). Gene length distribution showed that downregulated genes following cisplatin treatment were significantly longer, consistent with their higher probability to contain damage (Figure 5E,F).

Since downregulated genes could be a direct consequence of RNA polymerases blocked by cisplatin adducts, we focused on genes that were upregulated following cisplatin treatment. Using a minimum log_2_ fold change of 1 and a maximum adjusted *P* value of 0.05 as a cutoff, we found that more genes were upregulated in sensitive A2780 cells compared to resistant Acis cells (580 vs. 118 genes, respectively, Figure 5B,C, and Supplementary Tables S1–S6). The lower transcriptional response in resistant cells could be a consequence of the lower damage levels. Alternatively, higher basal expression of damage-responsive genes in resistant cells could be the underlying reason for the lower damage levels. We identified a list of 56 “primed” genes—genes that were upregulated after cisplatin treatment in A2780 cells, and were expressed at higher levels in Acis cells compared to A2780 cells prior to treatment (Figure 5G). Of these, 25 were previously reported as associated with the DNA damage response or cisplatin resistance (Supplementary Table S7). For seven of the primed genes, which had a basal expression level above a threshold of RPKM > 70 in Acis cells and that passed real-time PCR primer validation, we conducted quantitative real-time PCR to establish whether their expression was also upregulated in three additional cell lines: 293T (transformed human embryonic kidney cells), A549 (lung adenocarcinoma cancer), and U2OS (bone osterosarcoma) (Figure 5H). These genes were ARC, EGR2, MYLIP, OSGIN1, RHOV, TBR1, and SNAI1. While SNAI1 was previously reported as a cisplatin-responsive gene [29], the involvement of the other six genes in cisplatin response has not been previously reported. With the exception of one gene (OSGIN1), all the genes were upregulated in additional cell lines upon treatment with cisplatin. This strengthens the probability that they are indeed involved in the cisplatin response, making them attractive candidates for future studies.

## 2. Discussion

While cancer therapy is constantly improving and new personalized approaches develop, the use of systemic platinum treatment is still a first line of defense for many cancers, and cisplatin-resistance is still a major obstacle for successful recovery. There are multiple paths for obtaining cisplatin resistance, and therefore each experimental model may reflect distinct resistance mechanisms, and could be relevant for distinct cancer samples. To study a possible path to resistance, we used the model cancer cell lines of A2780 and their resistant derivatives, Acis, which share a genetic background. This allows us to pinpoint the changes associated with cisplatin resistance, in contrast to a study that would have compared sensitive and resistant cell lines from different sources.

Cisplatin kills cancer cells by damaging their DNA. From the DNA damage and repair perspective, similar to a previous report [30], our results show the resistant cell line accumulates less damage. This lower damage accumulation is not due to faster repair, as overall NER efficiency is similar in both resistant and sensitive cells. These results are consistent with recent study of oxaliplatin-sensitive and -resistant colon cancer cell lines [28]. As in lymphoblast and colorectal cancer cell lines, our genome-wide maps of cisplatin removal showed higher NER in transcribed and accessible regions of the genome [11,28,31]. We find that the balance between transcription-coupled and global NER was shifted towards transcription-coupled repair in the resistant cell line. A preference for transcription-coupled repair over global NER could be beneficial in coping with damage, as transcription-coupled repair will prioritize repair of transcribed regions, allowing cells to resume function faster. Furthermore, higher transcription-coupled repair could allow transcription to continue, explaining the attenuated transcriptional response to damage in resistant cells. However, we cannot conclude that this is a general mechanism for resistance since a parallel study in colorectal cancer cell lines actually reported an opposite shift, towards global genome repair in resistant cell lines [28].

Previous studies have shown that cisplatin damage formation is relatively uniform across the genome, dictated primarily by the frequency of target G-G dinucleotides [11,32,33]. Accessible regions in the genome are not associated with overall higher damage formation [11], but this and previous works show that repair is significantly enhanced at these regions [11,28]. Similar findings were reported for UV-induced damage [10,31,34]. Here, we measured alteration in chromatin accessibility three hours after cisplatin treatment, and did not find a significant shift in accessibility in either the sensitive or resistant cells. This is in contrast to a recent report that UV-treatment increases chromatin accessibility in skin fibroblasts [35]. In our hands, the transcriptional response to cisplatin is also more attenuated than the response to UV even in the same cell type (data not shown). These attenuated responses may reflect the different structure and properties of cisplatin- and UV- induced damage.

In general, repair, chromatin accessibility, and transcriptional profiles in the sensitive and resistant cell lines were very similar. Since NER efficiency did not appear to play a significant role in resistance, we focused on differences in gene expression that could drive the resistance phenotype. Studying gene expression changes in response to damage is complicated by the fact that cisplatin adducts block RNA polymerases. Thus, we focused on upregulated genes. The ability to compare differentially expressed genes in the resistant cell line, to a list of genes induced by cisplatin in the same genetic background allowed us to pinpoint 56 candidate “primed genes” implicated in resistance. Many of these primed genes were previously implicated in cisplatin or DNA damage response, but many are not yet characterized. Of the six previously unreported genes we tested, five showed increased expression after cisplatin treatment in additional cell lines. SNAI1, which was also on our list, has been associated with cisplatin response. Its disruption is associated with sensitivity and its overexpression with resistance to the drug [29,36,37,38]. Similar future studies into ARC, EGR2, MYLIP, RHOV, and TBR1, and other candidates from our list, will be beneficial to determine their involvement in cisplatin resistance, or DNA damage response.

There are currently over 1200 ongoing clinical trials involving cisplatin. (https://clinicaltrials.gov/ accessed on 28 May 2021). Many of these involve combination therapies geared towards overcoming resistance. Since cisplatin resistance is multifactorial, there are multiple avenues for overcoming it, recently reviewed in [14]. These include drugs that target DNA repair and cell cycle mechanisms that allow cancer cells to cope with cisplatin-induced damage. As new candidates for resistance are identified, new avenues for efficient, cancer-selective therapies can be developed to improve treatment and survival.

## 3. Materials and Methods

### 3.1. Cell Culture and Treatment

A2780 and Acis cells (Sigma-Aldrich 93112519 and 93112517, Rehovot, Israel) were grown in RPMI 1640 Medium (Biological Industries 01-100-1A, Beit Haemek, Israel) supplemented with 10% FBS (Gibco 10270106, Rhenium, Modi’in, Israel), 2 mM glutamine (Biological Industries 03-020-1B, Beit Haemek, Israel), 1 mM sodium pyruvate (Biological Industries 03-042-1B, Beit Haemek, Israel), 100 units/mL penicillin, 0.25 mg/mL streptomycin (Biological Industries 03-031-1B, Beit Haemek, Israel). Every three passages Acis cells were treated with 1 µM cisplatin (Pharmachemie BV, Teva group 031 30 25429 05, Haarlem, The Netherlands). 293T and A549 cells were grown in DMEM medium (Biological Industries 01-055-1A, Beit Haemek, Israel) supplemented with 10% FBS, 2 mM glutamine, 1 mM sodium pyruvate, 100 units/mL penicillin, and 0.25 mg/mL streptomycin. U2OS cells were grown in DMEM medium supplemented as described but without sodium pyruvate. Mycoplasma was monitored every 3–4 months.

### 3.2. UV and Cisplatin Treatment

For damage treatment, cells were grown to ~80% confluence in 60 mm or 100 mm dish, culture medium was removed, cells were washed once in PBS and were treated with either UV or cisplatin. For UV experiments cells were irradiated using UVC lamp (254 nm, UVP XX15S, 95-0042-09) with different doses according to the experiment. For cisplatin treatment, cells were incubated with media containing cisplatin at different doses. For repair experiments, media with cisplatin was removed after an initial incubation and replaced with fresh culture for the indicated time.

### 3.3. Cell Viability Assay

A2780 and Acis cells were seeded in 96-well plates at 10,000–14,000 cells per well. Cells were treated with different doses of either cisplatin or UV. Viability was measured 48 h post treatment using CellTiter-Glo^®^ Luminescent Cell Viability Assay Kit (Promega #G7571, Biological Industries, Beit Haemek, Israel) according to manufacturer’s instructions. Viability luminescent was measured using Cytation 3 Imaging Reader (BioTek, Winooski, VT, USA).

### 3.4. Immuno-Slot Blot Assay

DNA was extracted using PureLink Genomic DNA Mini Kit (Invitrogen, K1820-01). DNA (200–500 ng per well, two technical replicates per sample) was vacuum-transferred to a nitrocellulose membrane using the Bio-Dot or bio-dot SF apparatus (Bio-Rad, 1706542/5, Rishon LeZion, Israel). The membrane was baked at 80 °C for 90 min in a Bio-Rad’s Gel Dryer model 583, blocked in 5% milk, then incubated with damage-specific primary antibodies: anti 6-4 PP diluted 1:2000 (Cosmo bio, NM-DND-002, Otaru, Hokkaido, Japan), anti CPD diluted 1:4000 (Cosmo bio, CAC-NM-DND-001, Otaru, Hokkaido, Japan), and anti-cisplatin diluted 1:5000–1:15,000 (abcam, 103261, Cambridge, UK). After incubation with an HRP-conjugated secondary antibody, NA931 or NA935 (Cytiva, Marlborough, MA, USA) for UV damage and cisplatin damage, respectively, the damage signal was detected using Enhanced chemiluminescence (ECL™ Prime Western Blotting System, Cytiva, RPN2232, Marlborough, MA, USA). Genomic DNA amount loaded onto the membrane was quantified using SYBR™ Gold Nucleic Acid Gel Stain (Invitrogen, S11494, Carlsbad, CA, USA), and the damage signal was normalized according to SYBR-Gold signal using ImageJ software version 1.5.1j8 and Image Lab version 6.0 Bio-Rad.

### 3.5. Purification and Detection of Excision Products

The chemiluminescent excision assay was performed as described previously [39], with several modifications. In brief, culture medium was removed, cells were trypsinized and harvested in ice-cold media, centrifuged, and washed twice in ice-cold PBS. Cell pellets were resuspended in 400 μL lysis buffer (20 mM Tris-HCl pH 7.5, 150 mM NaCl, 1 mM EDTA, 1 mM EGTA, 1% Triton X-100), and incubated at 4 °C for 15 min on a rotary mixer. During the initial lysis, 50 fmol of a 50 nt oligomer was added as spike-in DNA for internal control. Low molecular weight DNA was isolated by centrifugation at maximum speed (16,873 g) for 30 min at 4 °C. The supernatant was treated with 1 µg/µL RNaseA (Sigma-Aldrich R4642, Rehovot, Israel) and 8 units/mL proteinase K (NEB, #P8107S, Ipswich, MA, USA) for 30 min at 55 °C. Samples were purified by phenol/chloroform extraction followed by ethanol precipitation. To remove high molecular weight DNA contaminants, 1.2X PCR cleanup beads (MagBio, AC-60005, Gaithersburg, MD, USA) were used for reverse size selection, followed by an additional ethanol precipitation. For biotin labeling, samples were incubated with 4 units of terminal deoxynucleotidyl transferase (NEB #M0315, Ipswich, MA, USA) and 20 μM biotin-16-ddUTP (Merck, 11427598910, Kenilworth, NJ, USA) for 1 h at 37 °C according to the manufacturer’s protocol. Biotinylation reaction was stopped by adding stop buffer (50 mM Tris pH 8, 10 mM EDTA, 0.4% SDS, 20 µg/mL glycogen). Biotin-labeled excision products were then precipitated with ethanol, resolved in a 12% urea-polyacrylamide gel, and transferred at 25 V for 30 min to hybond N+ nylon membrane (Amersham #RPN303B, Chalfont St Giles, UK) using semi-dry transfer (Bio-Rad Trans-Blot Turbo transfer system). DNA was crosslinked to the membrane using 150 mJ/cm^2^ in Hoefer UVC 500 crosslinker. The membrane was incubated for 30 min in TBS + 2% SDS buffer (50 mM Tris pH 7.4, 150 mM NaCl, 2% SDS) and then incubated in streptavidin HRP (abcam, ab7403, Cambridge, UK) diluted 1:10,000 in TBS + 2%SDS buffer for 1 h to achieve biotin–streptavidin binding. The membrane was sequentially washed twice for 5 min in each of the following buffers: TBS + 2% SDS, TBS + 0.05%SDS, and TBS +0.1% Tween-20, and finally another wash in 1XTBS. Biotin-labeled DNA signal was detected using enhanced chemiluminescence (ECL™ Prime Western Blotting System, GE Healthcare, RPN2232, Chalfont St Giles, UK).

### 3.6. RNA-seq

A2780 cells were seeded in 6-well plates at 6 × 10^5^ cells per well and Acis cells at 8 × 10^5^ cells per well in triplicates for each condition. Cells were incubated for 3 h with fresh media containing 200μM cisplatin. Total RNA was extracted using GENEzol™ TriRNA Pure with DNaseI Kit (Geneaid, GZXD, New Taipei City, Taiwan). RNA quality was assessed using RNA ScreenTape (Agilent, 5067–5576, Santa Clara, CA, USA) on Agilent 4200 TapeStation, all samples having 10.0 RINe score. Ribo-depleted RNA-Seq libraries were prepared using KAPA-Stranded RNA-Seq Kit with RiboErase HMR (Kapa KK8483, Cape Twon, South Africa) according to the manufacturer’s protocol, starting with 1 μg total RNA, and using Illumina TrueSeq adapters. Library quality and concentration was assessed using High Sensitivity RNA ScreenTape Analysis (Agilent, 5067–5579, Santa Clara, CA, USA).

All 12 libraries were pooled and sequenced in a single NextSeq 500 lane producing at least 17 million single-end reads per sample (75 nt long). Library quality was analyzed using FastQC version 0.11.8 (Babraham Bioinformatics). Reads were aligned to the genome using STAR V 2.7.3a [40]. PCR duplicates were removed using PicardCommandLine MarkDuplicates (http://broadinstitute.github.io/picard accessed on 28 May 2021). Read counts per gene were calculated using htseq-count v0.11.1 [41] with the command option: --stranded=reverse. DESeq2 was applied using Log2FC threshold of ±1 and adjusted *p*-value < 0.05, to identify differentially expressed genes for three conditions: A2780 vs. A2780 + cisplatin, Acis vs. Acis+cisplatin, and A2780 vs. Acis (Supplementary Tables S1–S6). Volcano plots for all three conditions were created using the EnhancedVolcano package [42]. The primed gene list (56 genes) (Supplementary Table S7) was created by intersecting the upregulated genes following cisplatin treatment in A2780 cells with the upregulated genes in Acis cells compared to A2780 cells. All plots were created using ggplot2 package [43].

### 3.7. XR-seq

XR-seq was performed as described in [11]. Briefly, 220 × 10^6^–290 × 10^6^ cells per library were harvested, collected by centrifugation and resuspended in lysis buffer and lysed using a Dounce homogenizer. Low molecular weight DNA was isolated, and samples were incubated with RNase A (Qiagen, 1007885). Primary excision products were pulled down by TFIIH co-immunoprecipitation using anti-p62 antibody (Santa-Cruz Biotechnology, sc-293) and anti-p89 antibody (Santa-Cruz Biotechnology, sc-292). Adapters were ligated on both ends of the excision oligomer and immunoprecipitated using cisplatin-specific antibody (abcam, 103261, Cambridge, UK). Cisplatin-induced damages were reversed in vitro by incubating the samples with 200 mM NaCN at 65 °C overnight. DNA was PCR amplified and purified by native polyacrylamide gel electrophoresis (PAGE) to create the library. Library quality was assessed using Agilent 4200 TapeStation.

Libraries were pooled and sequenced on a HiSeq 2500 Illumina sequencer. Quality score for each nucleotide was analyzed using the fastx-toolkit to ensure only high quality reads were processed. The adapter sequence was trimmed from each read using Trimmomatic [44] version 0.36 with the command options: ILLUMINACLIP:adapter_sequence.txt:2:30:10. Following adapter removal, 50 nt length reads were filtered. Reads were aligned to the genome using Bowtie [45] with the command options: -q --nomaqround --phred33-quals -p 32 -m 4 -n 2 -e 70 -l 20 --chunkmbs 800 --best –S. For each sample we obtained 4.6–7.8 million unique aligned reads. Following alignment, reads mapped to chromosome Y or mitochondrial chromosome were filtered and PCR duplicates were removed using PicardCommandLine MarkDuplicates.

For replicate correlation plots, a 700 bp windows bed file was created using bedtools makewindows. Read counts over these windows was calculated for each replicate using bedtools coverage and Pearson correlation coefficients were calculated using the corrplot R package.

For correlation with RNA-Seq data, read counts per gene were calculated using htseq-count v0.11.1 [41] for both experiments and Spearman correlation plots were plotted using R.

To plot average XR-seq signal along genes, the gene’s annotation file was downloaded from Ensembl, assembly GRCh38, release 96. Non-overlapping regions around the TSS were obtained using custom scripts and BEDTools (version 2.27.1, [46]) slop and merge commands. Only genes that had RPKM > = 10 in A2780 or Acis cells were included in the plot. All samples were converted to BED format using bedtools bamtobed command. Reads intersecting gene list were obtained using bedtools intersect command and strand-specific profiles over the TSS were created using the R (version 4.0.0.) Bioconductor genomation package (version 1.20.0, [47]). Read counts for each gene interval were obtained using bedtools coverage command, and plotted using ggplot.

### 3.8. ATAC-seq

ATAC-seq was performed using an ATAC-seq kit (Active motif, 53150, Carlsbad, CA, USA) according to the manufacturer’s protocol. Prior to ATAC-seq, cells were washed with PBS and incubated in the 37 °C incubator with media containing 200 units/mL DNase (Worthington, LS002006) in 1.2 mM MgCl_2_ and 0.5 mM CaCl_2_ for 30 min. For each reaction 100,000 cells were taken. ATAC-seq libraries were purified and size selected with HighPrep PCR beads (MagBio, AC-60005, Gaithersburg, MD, USA). Library quality was assessed using Agilent 4200 TapeStation.

Libraries were pooled and sequenced using on an Illumina NextSeq500 sequencer. Sequencing quality was assessed using FastQC (version 0.11.8). The adapter sequence was trimmed from each read using Cutadapt [48] (version 1.15) with the command options: -m 10. Reads were aligned to the genome using Bowtie2 [49] with the command options: --very-sensitive -k 10. For each sample we obtained at least 9 million unique aligned reads. Following alignment, reads that were mapped to chromosome Y or mitochondrial chromosome were filtered and PCR duplicates were removed using PicardCommandLine MarkDuplicates (version 2.23.4). Insert size distribution histograms were plotted using deeptools (version 3.5.0) bamPEFragmentSize command (nucleosome free region and mononucleosome peak were confirmed in each sample). All bam files of replicates from each condition were merged to create a pooled bam file. Peaks were called separately for each replicate and pooled bam files using macs2 callpeak command (version 2.2.7.1 [50]). Peak files from replicates of the same condition were merged using bedtools intersect to create one set of peaks per condition. Peak regions were chosen only if there was more than 50% overlap of the peak region between all replicates per condition. For replicate correlation plots, a union set of all four conditions’ peaks was created, read counts over these regions was calculated for each replicate using bedtools coverage, and Pearson correlation coefficients were calculated using the corrplot R package.

### 3.9. UCSC Track Visualization

All replicate bam files were merged using samtools merge command. Per base read counts were calculated using bedtools genomecov and normalized per billion mapped reads per chromosome. Read counts were calculated in windows using awk (RNA-Seq: 75 nt windows, XR-Seq: 25 nt windows, ATAC-Seq: per base coverage), and converted to bigwig using the bedGraphToBigWig command to create signal maps in bigwig file format.

### 3.10. Real-Time PCR

Total RNA was extracted with GENEzol™ TriRNA Pure Kit (Geneaid, GZXD, New Taipei City, Taiwan) and cDNA prepared using qScript cDNA Synthesis Kit (QuantaBio 95047, Beverly, MA, USA). Expression level of primed genes was determined by Real-time PCR conducted on a Bio-Rad CFX96 or CFX384 systems using iTaq Universal SYBR Green Supermix (Bio-Rad 1725121, Rishon LeZion, Israel). Primers sequences are shown in Supplementary Table S8.

## Data Availability

XR-seq, RNA-seq, and ATAC-seq data files are available in GEO under accession GSE173201. Data can be viewed in the USCS genome browser by pasting the following link as a track hub: https://data.cyverse.org/dav-anon/iplant/home/hadargo/A2780_Acis_track/hub.txt (accessed on 28 May 2021).

## Supplementary Materials

The supplementary materials are available online at https://www.mdpi.com/article/10.3390/ijms22115814/s1.
