# Supplementary material for: Genomic Characterization of Cisplatin Response Uncovers Priming of Cisplatin-Induced Genes in a Resistant Cell Line
Source: Int J Mol Sci. 2021 May 28;22(11):5814. doi: 10.3390/ijms22115814 (PMC8198185; doi:10.3390/ijms22115814)
Supplement: Supplementary file 1 [file ijms-22-05814-s001.zip › Supplementary Figures.pdf]

Supplementary Figures

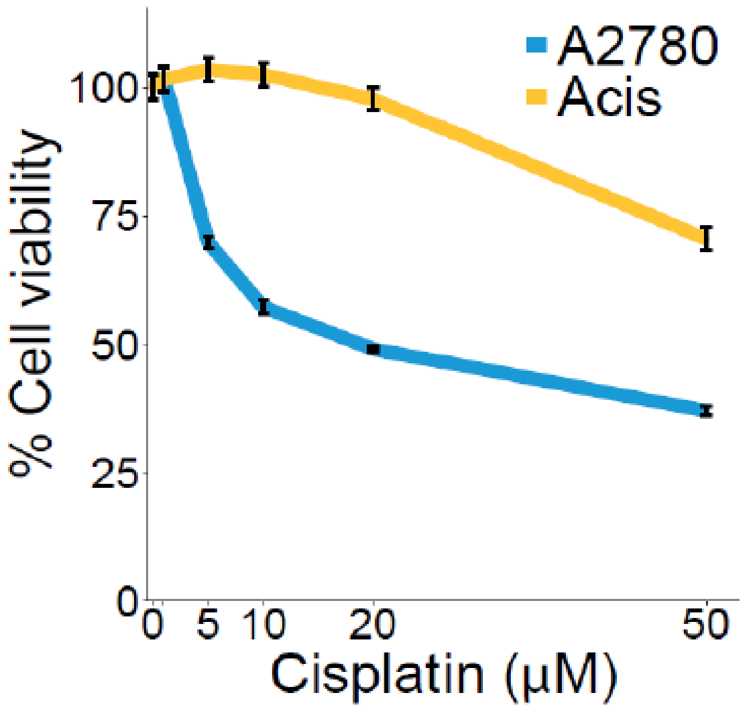

Figure S1. cell viability of A2780 (cisplatin sensitive) and Acis (cisplatin resistant) cell lines following increasing cisplatin doses.

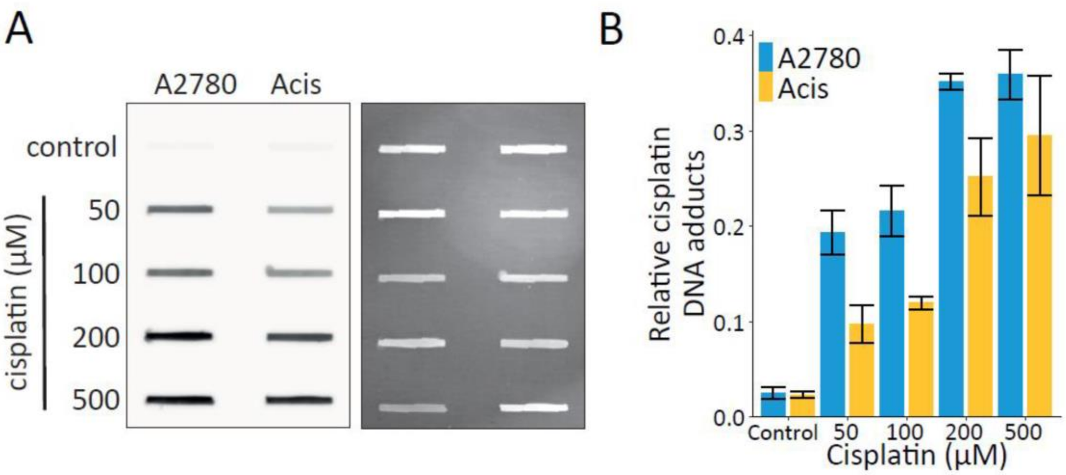

Figure S2. (**A**) Representative cisplatin initial damage signal detected by immuno-slot blotting (left panel) following increasing cisplatin doses treatment (50µM–500µM). Total nucleic acid amounts measured by SYBR-Gold staining (right panel). (**B**) Damage signal normalized to total nucleic acid amounts. Average and error bars (SEM) are shown for data from two biological replicates.

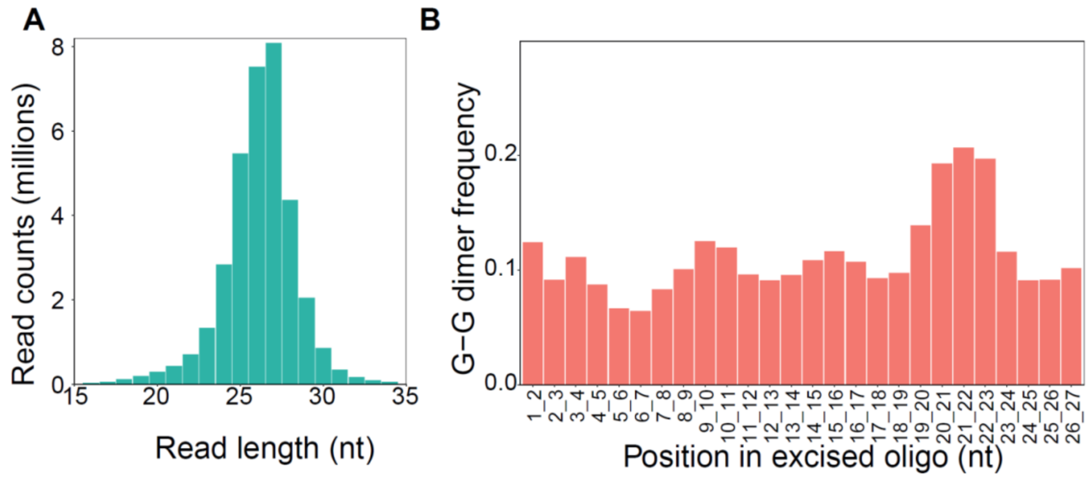

Figure S3. XR-Seq data quality control. (**A**) Representative distribution of excised oligonucleotide sequence lengths, calculated after removal of flanking adapter sequences from sequences 50-nt reads. (**B**) Representative frequency of the G-G dinucleotide at each position of the 27-nt-long XR-Seq excision fragments. Data presented from replicate 1 of A2780.

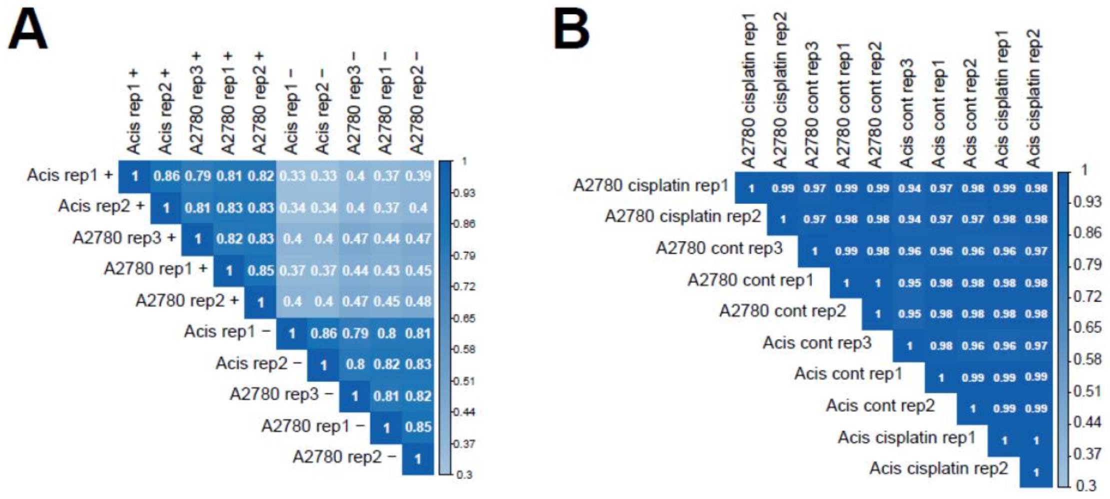

Figure S4. (**A**) Pearson correlation between cisplatin XR-Seq libraries calculated over 700 bo windows. (**B**) Pearson correlation between ATAC-Seq libraries calculated over union set of peaks from all four conditions.
